# Prospective Study of Dot Phrase Instructions to Improve Quality and Timeliness of Bowel Preparation for Inpatient Colonoscopies

**DOI:** 10.7759/cureus.74040

**Published:** 2024-11-19

**Authors:** Michael Kurin, Syed Ahmad Adil, Roma Patel, Muhammed Alikhan, Abbinaya Elangovan, Alok Tripathi, Mayada Ismail, Sagarika Satyavada, Raj Shah, Gregory Cooper

**Affiliations:** 1 Gastroenterology, University Hospitals Cleveland Medical Center, Cleveland, USA; 2 Internal Medicine, University Hospitals Cleveland Medical Center, Cleveland, USA

**Keywords:** colonoscopy bowel preparation, diabetes mellitus type 2, dot phrase, inpatient colonoscopy, split-dose prep

## Abstract

Background

Inpatient bowel preparation is often suboptimal. Few interventions have been effective at improving its success rate. We determined the clinical features associated with suboptimal inpatient bowel preparation and analyzed the ability of an easily implementable set of instructions inserted into the electronic health record to improve the success of bowel preparation.

Methods

We prospectively collected bowel preparation outcomes, demographics, and clinical features for inpatient colonoscopies in a tertiary center from July to November 2019, forming the standard of care (SOC) arm. After introducing a standardized set of instructions into the electronic health records using a “dot phrase,” data for the intervention arm was collected from December 2019 to May 2020. We compared the outcomes of preparation quality and efficiency between these groups. We calculated avoidable charge estimates to determine the cost savings of our intervention. Groups were combined and multivariate analysis was performed to determine the clinical features independently associated with suboptimal bowel preparation.

Results

Sixty-eight patients were included in the SOC arm and 76 in the intervention arm. Post-intervention, there was a 36.6% reduction in the number of patients with a suboptimal bowel preparation outcome, though multivariate analysis did not show an independent association with optimal bowel preparation. In the multivariate analysis, only diabetes mellitus (p = 0.046) was independently associated with suboptimal preparation.

Conclusions

Diabetes mellitus is a non-modifiable risk factor for suboptimal bowel preparation. Our standardized proactive instructions for inpatient bowel preparation administration led to a modest reduction in insufficient bowel preparation. This could lead to significant cost savings.

## Introduction

Suboptimal bowel preparation (prep) is one of the greatest challenges of inpatient colonoscopies. Previous studies have reported a 20-30% rate of suboptimal prep in the general population and inpatient status has consistently proven to be an independent risk factor for inadequate bowel prep [1]. Other studies have reported a 50-75% rate of suboptimal prep in inpatients alone [2-4]. While the reason for decreased prep quality in inpatients is not completely understood, the cause is likely multifactorial. Inpatients may be acutely ill and are likely to have more comorbidities and less mobility than outpatients, which may decrease gut motility and limit the efficacy of bowel prep, limit the volume of prep they can tolerate, and affect their ability to follow directions [5]. Furthermore, reliance on busy house staff and nursing to administer the prep may come with less individual attention than the outpatient would receive from family members [6]. Non-modifiable risk factors for inadequate inpatient bowel prep that have been identified include a history of constipation, decreased mobility, diabetes mellitus (DM), use of antipsychotic medication, and prolonged hospital stay [7]. Modifiable factors that have been associated with suboptimal inpatient bowel prep include limitations in patient education and instruction, and the lack of a structured and standardized bowel prep administration plan [8]. The use of constipating medications, especially narcotic pain medications, and uncontrolled nausea are also likely more prevalent among inpatients and may affect the efficacy of the bowel prep and the patient’s tolerance of it, respectively.

There are two ways in which inpatient bowel prep can be suboptimal. The patient’s stool is deemed insufficiently clear before attempting the colonoscopy, necessitating a delay to allow more prep consumption. This could have occurred due to incomplete consumption of the recommended amount of bowel prep, or inefficacy of the prep. Alternatively, bowel prep can be suboptimal when the procedure is attempted but there is inadequate visualization of the mucosa due to incomplete clearance of stool from the colon. In this paper, we will use the term "inadequate bowel prep" to describe the latter phenomenon, while the terms "incomplete," or "insufficient" bowel prep consumption will be used to describe the former. The term "suboptimal" bowel prep will be the umbrella term to include both phenomena.

The primary aim of this study is to design and proactively test the effectiveness of a standardized set of proactive instructions that address modifiable barriers to optimal bowel prep. The second aim is to identify risk factors that contribute to suboptimal inpatient bowel prep, whether it be delays in the start time of procedures due to insufficient bowel prep, inadequate prep quality, or both. 

## Materials and methods

Patient selection and data collection

All patients who underwent inpatient colonoscopy at a US tertiary medical center from July 2019 to May 2020 were eligible for inclusion. Patients who received bowel prep through a feeding tube, received rapid prep for an emergent colonoscopy, and patients who had been planned in advance for a two-day prep were excluded. A power analysis was conducted using an online calculator (Sample Size Calculator (clincalc.com)), for sample size estimation. Based on the available literature and the fact that our criteria for optimal prep are more stringent as they incorporate both adequacy of prep and lack of procedural delays, we predicted a 45% rate of suboptimal prep for the standard of care (SOC) arm. Using significance criteria of alpha=0.05 and power=0.80, with an estimated absolute decrease in suboptimal prep of 25%, the minimum sample size required to detect this difference is 108.

Intervention

**Figure 1:**
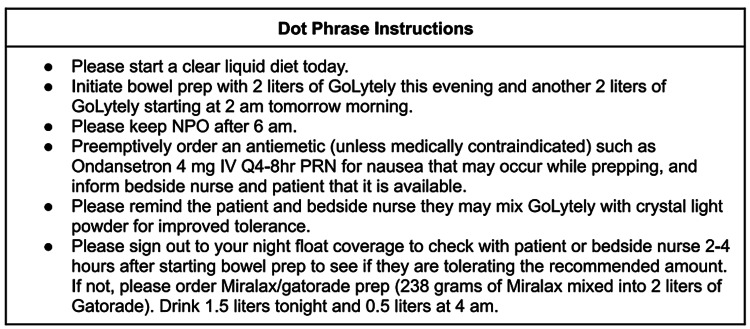
Dot Phrase Instructions

From July to November 2019, we included patients who received the SOC, meaning consulting gastroenterologists could continue to give recommendations for bowel prep in the usual fashion without any additional instruction. At our institution, this typically includes standard recommendations for a clear liquid diet the day prior to the procedure, and split-dose bowel prep with 4 L of polyethylene glycol (PEG) (GoLytely®). Patients who had a colonoscopy recommended during this time period made the SOC arm. From December 2019 to May 2020, all consulting gastroenterologists were asked to use a standardized dot phrase for bowel prep instruction in their consult note the day prior to the colonoscopy (Figure 1). A dot phrase is a preformed set of text that is easily inserted into a note in the electronic health record by typing a short code preceded by a dot. In addition to the standard split-dose GoLytely bowel prep, the dot phrase instructed inpatient teams to ask their night coverage to determine whether the patient is tolerating their prep the evening prior to the procedure, and if not outlines an alternative better-tolerated purgatory split-dose formula, 238 g PEG (Miralax®) in 2 L sports drink, which should be implemented instead. It also advises proactive ordering of as-needed antiemetic medications unless medically contraindicated and instructs the team to communicate the potential need for antiemetics with the bedside nurse and the patient in advance. Implementation of this intervention was advertised to internal medicine residents via a one-time email from the chief medical residents at the beginning of December 2019. Patients who had a colonoscopy recommended during this time period made up the intervention arm. 

Data collection

For all included patients in both arms, we used a medical record review to collect demographics, body mass index (BMI), medical comorbidities, and medication history. We documented the type of diet consumed the day prior to the colonoscopy, the type of bowel prep consumed, amount of prep consumed, duration of bowel prep, incidence of nausea or vomiting during prep consumption, use of antiemetics during the bowel prep, use of narcotics within 48 hours prior to the procedure, and delays in procedure performance related to perceived insufficient bowel prep consumption. When documenting delays in procedure performance, we also specified whether there was a partial day delay, meaning the procedure was delayed from morning to afternoon to allow for more time to complete the bowel prep, or a delay to the next calendar day. The number of calendar days the procedure was delayed was also documented in cases when there was a delay of more than one day. We also documented the length of hospital stay, defined as the number of days elapsed from the date of admission to the date of the colonoscopy. Once the procedure was attempted, we documented, in real-time, the relevant procedural outcomes including the Boston Bowel Prep Score (BBPS) if documented by the endoscopist, the qualitative description of the quality of the bowel prep as documented by the endoscopist, and whether the procedure was completed in entirety.

Data analysis

To determine the efficacy of the dot phrase, the procedural outcome measures including BBPS, qualitative description of prep quality, and prep-related procedural delays, as well as incidence of nausea and antiemetic use during the bowel prep were compared between the SOC and the intervention arms. Patient age, BMI, narcotic use, history of constipation, and history of diabetes were also accounted for using multivariate logistic regression analysis to ensure a lack of confounders.

To determine the factors associated with delay in completion of bowel prep and inadequate bowel prep, patients from both the SOC and intervention arms were analyzed in aggregate. The patients were then divided into three groups:

● Patients who were determined to have inadequate bowel prep during colonoscopy.

● Patients who required a delay of their procedure due to insufficient bowel prep.

● Patients who did not require any delay and had adequate bowel prep.

The first two groups were aggregated and duplicates were removed to form the group of patients who either required delay of their procedure, had inadequate bowel prep, or both. This aggregated group was compared against the third group whose bowel prep process proceeded optimally. Non-modifiable factors such as demographics, BMI, medical comorbidities, and medications, as well as potentially modifiable factors such as type of bowel prep consumed, amount of prep consumed, incident nausea or vomiting during the bowel prep, in-hospital narcotic use, and duration of hospital stay prior to colonoscopy were compared between these two cohorts. The same comparison was then made between all patients who required a delay of procedure for insufficient bowel prep vs. patients whose procedures went on schedule. Finally, the same comparison was again made between patients who had an inadequate bowel prep as determined by the endoscopist vs. patients with an adequate or better bowel prep quality. Continuous variables are reported as mean ± standard deviation or median with interquartile range. Categorical data were compared using Fisher’s exact test. Continuous data were compared using the Mann-Whitney U-test for a two-tailed alpha. Statistical significance was presumed for p <0.05. IBM SPSS (version 28.0; IBM Corp, Armonk, NY) was used for statistical analysis. 

In addition to the above clinical outcome comparisons, we performed a cost-savings analysis to determine whether the use of dot phrase decreased the amount of avoidable healthcare charges incurred due to challenges of bowel prep. Avoidable charge estimates were defined as the sum of the colonoscopy with anesthesia fee (including technical and professional charges for colonoscopy with anesthesia) as obtained from our hospital billing department, and the average charge for one day of room and board in a hospital, obtained from www.healthcarebluebook.com. The colonoscopy with anesthesia fee was determined to be $4675 per procedure. The charge for one day of room and board was $1800. To obtain the total avoidable charge estimate, we multiplied the number of additional colonoscopies required due to inadequate prep during the inpatient procedure by $4675 and added this value to the total number of avoidable hospital days incurred due to delays in completion of bowel prep, multiplied by $1800.

The study was conducted after approval by the Institutional Review Board (IRB) at our institution.

## Results

One hundred and forty-four patients were included in the study, 68 patients in the pre-intervention period and 76 patients in the post-intervention period. During the post-intervention period, the proactive bowel prep instructions were included in the electronic notes by the gastroenterology team in 58 out of the 76 patients who underwent colonoscopy. Using an intention-to-treat (ITT) approach, there was a 36.6% reduction in the number of patients with insufficient prep requiring procedural delay or inadequate bowel prep in the post-intervention cohort (p = 0.04) (Table 1). However, multivariate analysis did not show the use of the dot phrase to be an independent association with a reduction in insufficient prep (Table 2). Using per-protocol analysis, whereby only those patients for whom the standardized instruction set was input into the electronic medical record were included in the post-intervention group, there was a 32.8% reduction in the number of patients with delay or inadequate bowel prep in the post-intervention cohort (p = 0.16) (Table 1). There was no significant difference between the pre- and post-intervention cohorts in terms of age, gender, or length of hospital stay. The two cohorts were similar in the number of patients who had either colitis or DM. There was no significant difference in the percentage of patients with reported nausea or the percentage of patients with antiemetic use between the two cohorts (Table 1). 

**Table 1:** Efficacy of Dot Phrase BBPS: Boston Bowel Prep Score; NS: nonsignificant.

	Intention to Treat			Per Protocol		
	Pre-Intervention (N = 68)	Post-Intervention (N = 76)	p-Value	No Dot Phrase (N = 86)	Used Dot Phrase (N = 58)	p-Value
Inadequate Preparation, N (%)	16 (23)	12 (16)	NS	19 (22)	9 (16)	NS
At Least One Day Delay, N (%)	8 (11)	5 (7)	NS	8 (9)	4 (7)	NS
Delay to Afternoon, N (%)	12 (17)	7 (9)	NS	13 (15)	6 (10)	NS
Total Days Delayed (days)	10	5		8	4	
Any Delay or Inadequate Preparation, N (%)	31 (46)	21 (28)	0.04	35 (40)	16 (28)	0.16
BBPS Mean (Std Dev)	7.0 (1.9)	7.4 (1.3)	NS	7.2 (1.7)	7.2 (1.4)	NS
Nausea, N (%)	12 (17)	9 (12)	NS	12 (14)	9 (16)	NS
Antiemetic Use, N (%)	7 (10)	6 (8)	NS	7 (8)	6 (10)	NS
Colitis	3 (4)	3 (4)	NS	4 (5)	2 (3)	NS
Diabetes Mellitus, N (%)	17 (24)	18 (24)	NS	19 (22)	16 (28)	NS
Estimated Avoidable Healthcare Charges ($)	92,800	65,100		103,225	49,275	
Proportional Estimated Avoidable Healthcare Charges ($ per person)	1326	857		1,186	850	

**Table 2:** Logistic Regression Analysis BMI: body mass index. *For per-protocol analysis, replace with “dot phrase used.” **For per-protocol analysis, replace with “dot phrase not used.”

Clinical Feature		Odds for Inadequate Preparation			
		Intention to Treat		Per Protocol	
		OR (CI)	p-Value	OR (CI)	p-Value
Gender	Male	0.41 (0.18- 0.94)	0.034	0.39 (0.17-0.90)	0.026
	Female				
Age as Continuous		0.983 (0.954-1.013)	0.258	0.98 (0.95-1.01)	0.231
Narcotics	Yes	2.13 (0.86-5.30)	0.103	2.25 (0.91-5.57)	0.08
	No				
Constipation	Yes	1.09 (0.34-3.46)	0.881	1.02 (0.33-3.21)	0.97
	No				
Diabetes	Yes	2.72 (1.02-7.27)	0.046	2.71 (1.02-7.23)	0.046
	No				
BMI as Continuous		1.011 (0.964-1.06)	0.65	1.00 (0.96-1.06)	0.653
Pre-intervention*		0.74 (0.32-1.72)	0.49	1.04 (0.44-2.47)	0.931
Post-intervention**					

In the ITT analysis, the estimated avoidable healthcare charges were $92,800 in the pre-intervention group and $65,100 in the post-intervention group (Table 1). In the per-protocol analysis, the estimated avoidable healthcare charges per person were $1,186 in the group that did not utilize the dot phrase and $850 in the group that utilized the dot phrase (Table 1).

Of the total study population of 144 patients, 28 had inadequate prep and 19 had insufficient prep. After aggregating these groups and eliminating duplicates, a total of 40 unique patients had suboptimal prep (27.8%), which was defined as either inadequate or insufficient prep. When comparing patients with suboptimal prep to the 84 patients whose bowel prep went optimally, history of DM (p = 0.01), colitis (p = 0.02), and hyperthyroidism (p = 0.04) were found to be associated with suboptimal prep. On multivariate analysis, female gender was also found to be independently associated with suboptimal bowel prep. All other comorbidities investigated were not significantly different between the two groups (Table 3). Narcotic use trended toward an association with suboptimal prep, but this association did not meet statistical significance (p = 0.06). There was no significant difference in the amount of bowel prep consumed, the reported incidence of nausea, or the use of antiemetics among the cohorts (Table 4). 

**Table 3:** Demographics and Comorbidities Associated With Inadequate or Incomplete Bowel Preparation CHF: congestive heart failure; BMI: body mass index; CVA: cerebrovascular accident; HTN: hypertension; CAD: coronary artery disease; ESRD: end-stage renal disease; COPD: chronic obstructive pulmonary disease; OSA: obstructive sleep apnea; GI: gastrointestinal; NS: nonsignificant.

	Delay or Inadequate (N = 40)	Neither Delay nor Inadequate (N = 104)	p-Value
Demographic Features			
Age (Std Dev)	59.0 (13.8)	63.7 (14.1)	NS
Gender Male; N (%)	21 (48.8)	59 (66.3)	0.06
Caucasian; N (%)	20 (47.6)	55 (62.5)	0.2
African American, N (%)	19 (45.2)	33 (37.5)	0.2
Mean BMI (Std Dev)	29.8 (9.5)	29.1 (8.4)	NS
Comorbidities			
Diabetes	19 (43.2)	18 (20.7)	0.01
CHF	14 (31.8)	21 (24.1)	0.4
CVA	4 (9.1)	5 (5.7)	NS
HTN	25 (56.8)	48 (55.2)	NS
CAD	10 (22.7)	24 (27.6)	NS
Substance Use Disorder	5 (11.3)	4 (4.6)	0.2
Mood Disorder	8 (18.2)	12 (13.8)	NS
Hyperthyroidism	3 (6.8)	0 (0)	0.04
Hypothyroidism	4 (9.1)	11 (12.6)	NS
ESRD	5 (11.3)	9 (10.3)	NS
COPD	7 (15.9)	14 (16.1)	NS
OSA	5 (11.3)	9 (10.3)	NS
Colitis	6 (13.6)	2 (2.3)	0.02
Diverticulosis	3 (6.8)	8 (9.2)	NS
History of GI Bleed	6 (13.6)	5 (5.7)	0.2
Constipation, N (%)	5 (10.6)	19 (22.9)	NS

**Table 4:** Clinical Features Associated With Inadequate or Incomplete Bowel Preparation Std dev: standard deviation; NS: nonsignificant.

	Delay or Inadequate (N = 40)	Neither Delay nor Inadequate (N = 104)	p-Value
Amount Prep Consumed (L) (Std Dev)	4.6 (1.6)	4.0 (1.2)	NS
Nausea, N (%)	9 (19.1)	13 (13.8)	NS
Antiemetic Use, N (%)	6 (12.8)	7 (7.4)	NS
Median Length of Stay (Days)	6	5	NS
Psych Med Use, N (%)	15 (31.9)	24 (27.6)	NS
Narcotic Use, N (%)	17 (37.8)	19 (21.1)	0.06

## Discussion

The effects of inadequate bowel prep include decreased mucosal visualization leading to missed lesions, the need for repeat procedures, extended hospital stays, and overall increased costs [9,10]. Similarly, insufficient bowel prep consumption can be expected to delay procedures, extend hospital stays, increase costs, and affect patient and provider satisfaction. Attempts at improving inpatient bowel prep remain a work in progress.

Our prospective study is one of a few in the United States to test the effectiveness of a preemptive management strategy using standardized instructions for bowel prep, communicated via a dot phrase in gastroenterology consult notes, in a center already routinely using split-dose bowel prep. We are also one of the first to use a combination of prep quality and efficiency as the primary outcome of the study. In the ITT protocol, the post-intervention cohort had a significant reduction in insufficient prep, supporting the success of our intervention. When limiting the post-intervention cohort to those for whom the dot phrase was used, the reduction in suboptimal prep did not meet statistical significance. Multivariate analysis also showed no independent benefit to the use of the dot phrase after controlling for confounders. The primary reason for the lack of statistical significance is likely the sample size. Our power calculations assumed a large improvement in bowel prep, reducing the rate of insufficient prep from 45% to 20%. A larger sample size would likely have shown significant results for a more modest improvement. Additionally, there are likely several other factors that contributed to our intervention yielding only a modest improvement. First, the integration of the "dot phrase" into the electronic health record was accomplished for just 58 out of the 76 patients. This implies consulting gastroenterologists did not always remember to implement the "dot phrase." Furthermore, implementation of the instructions in the "dot phrase" required the primary service to read the instructions, place the appropriate orders, and communicate them to the patient and the bedside nurse. Outcomes were also dependent on the adherence of the bedside nurse to the instructions, which could not be assessed in this study but was likely imperfect. This is especially true for the last two months of our recruitment period, which coincided with the beginning of the COVID-19 pandemic. During that time period, bedside nurses and all healthcare providers likely spent less time at the patient's bedside and would have been less present to assist the patient with his or her bowel prep. In future studies, this challenge could be improved upon by involving representatives from bedside nursing in the process of creating the dot phrase, or by using a communication order directed to the bedside nurse rather than relying solely upon the primary team to communicate the recommendations from the consult note. Additionally, one important component of the "dot phrase" was the utilization of as-needed antiemetics to combat the effects of nausea brought on by the prep. Fewer patients than we expected reported nausea from the bowel prep and few made use of the available as-needed antiemetics. It is possible that nurses and patients were not made aware of the availability of antiemetics and that nausea was underreported, or it may be that nausea was not as important of a factor in tolerance of bowel prep as we predicted. Next, it is possible that some of the recommendations proposed in the "dot phrase" have already been incorporated into the practices of the SOC group, potentially contributing to the null outcomes observed. Despite the small sample size, there was a modest financial benefit for the hospital in the post-intervention period. This may be magnified as well in a larger study.

Our findings appear to contradict other recent studies that have shown greater benefit to similar interventions, such as a bowel prep order set or checklist. Educational interventions such as instructional booklets and videos have been shown to improve the quality of bowel prep in some small studies in addition to improving compliance and patient satisfaction [11-13]. However, testing the efficacy of these interventions has been limited by small sample sizes and poor reproducibility. In a 2018 study by Yadlapati et al., use of an order set for inpatient bowel prep using split-dose bowel prep along with routine assessment of prep quality, use of as-needed antiemetics, and availability of rescue laxatives demonstrated an improvement in proportion of adequate bowel prep from 42.5% to 85.7% (p < 0.001), a decreased length of post-prep-initiation hospital stay from 8.0 to 6.9 days (p = 0.02), and an annual cost savings of $46,000, though the proportion of delayed procedures due to insufficient bowel prep did not decrease (7.4% vs. 6.5%; p = 0.6) [14]. However, the control group in this study used single-dose prep, rather than split dose, and therefore this benefit may simply be due to the use of split-dose vs. single-dose bowel prep. This notion is further supported by a 2020 quality improvement study by Sullivan et al., which used an electronic order set consisting of a checklist with instructions for administering and monitoring inpatient colonoscopies using split-dose bowel prep, but only found significant improvement in the adequacy of prep in the right colon (75.0% vs 86.9%, p = 0.04) but not overall prep quality (73.7% vs 80.3%, p = 0.22) [2]. Since the split-dose regimen is thought to specifically improve the quality of prep in the right colon, the benefit found in this study may suggest that the use of a split dose rather than a single-dose regimen is the primary reason for improvement rather than the standardized checklist itself. Thus, whether the use of standardized and anticipatory instructions would offer further benefit in centers that already administer split-dose bowel prep regimens remains unknown. This is the question we attempted to address in our study.

Our study also determined the risk factors for suboptimal bowel prep in our center. We found that a history of DM is associated with suboptimal bowel prep in inpatients on multivariate analysis. These findings are consistent with the previous studies that have looked at risk factors associated with suboptimal prep. In a multicenter, prospective observational study of 1032 patients from Italy by Fuccio et al., DM (OR, 1.61; 95% CI, 1.18-2.20), use of antipsychotic drugs (OR, 3.26; 95% CI, 1.62-6.56), and seven or more days of hospitalization (OR, 1.02; 95% CI, 1.00-1.04) were associated with an increased risk of inadequate colon cleansing [7]. The exact mechanism behind suboptimal prep in diabetic patients is not completely understood, but it likely involves neuropathic intestinal dysmotility. In our study, neither antipsychotic use nor length of hospital stay was associated with any statistically significant difference in prep quality. This may be secondary to differences among population groups between the United States and Italy, or it could be that our smaller study was underpowered for these variables. Another retrospective study by Agrawal et al. found inpatient opioid use to be a predictor of inadequate bowel prep among a cohort of 1029 patients at a tertiary care hospital (OR, 1.69; p = 0.04) [15]. In our study, inpatient opioid use trended toward inadequate bowel prep but did not meet statistical significance (p = 0.06).

Our study has several limitations. This is a single-center study involving a tertiary academic medical center and the results may not be generalizable. One potential bias is the Hawthorne effect among endoscopists tasked with assessing the quality of the patient’s prep within the intervention group. However, we do not believe that this played a significant role as most endoscopists were not aware of the details of the study at the time of endoscopy. The use of a dot phrase that was dependent on others for implementation rather than a true order set introduced the variable of provider adherence that could have detracted from the potential benefits of the protocol. As mentioned above, the small sample size likely prevented finding more statistically significant outcomes. The strengths of our study include prospective data collection, the use of a robust primary outcome that combines prep quality with prep efficiency, based on a need for prep-related procedure delay, and the use of multivariate analysis to confirm factors independently associated with our primary outcome.

Inpatient colonoscopy continues to be a major challenge to healthcare systems and continues to contribute to wasted resources and costs. While our study found that the addition of a standardized dot phrase of anticipatory guidance for those ordering and administering inpatient bowel prep was not independently associated with a reduction in insufficient bowel prep, it did demonstrate that even modest improvements in prep quality and efficiency could lead to substantial cost savings. Further studies using an order set rather than written instructions and a larger sample size are needed to more definitively determine whether such anticipatory guidance can lead to significant improvements in inpatient bowel prep. While our study did not directly incorporate patient participation in the evaluations of their bowel prep, interventions are poised to yield more substantial outcomes through active patient involvement. In light of the dynamic healthcare landscape, leveraging sophisticated portals or applications designed to enhance patient engagement could present a promising avenue for enhancing inpatient bowel prep. Our study supported recently published data that a history of DM is one of the most important features associated with suboptimal inpatient bowel prep. Narcotic use and female gender may contribute as well. By identifying these risk factors, the study can help inform future interventions aimed at reducing the incidence of delayed procedures and optimizing the bowel prep process in an individualized, patient-centered way.

## Conclusions

In summary, suboptimal inpatient bowel prep is linked to reduced patient satisfaction and increased costs. While standardized order sets have shown promise, this study highlights the effectiveness of a cost-free "dot phrase" intervention in the electronic health record. This intervention improves bowel prep, even in patients already on a split-dose regimen, resulting in notable cost savings. Additionally, while nausea was less commonly reported as a barrier to prep quality, DM and female gender emerged as significant risk factors. These findings underscore the need for tailored interventions to improve inpatient bowel preps.
